# Editorial: Ion channels in the nervous system

**DOI:** 10.3389/fncel.2026.1812138

**Published:** 2026-03-17

**Authors:** Janire Urrutia, Alvaro Villarroel, Miren Revuelta

**Affiliations:** 1 Department of Physiology, Faculty of Medicine and Nursery, University of the Basque Country, Leioa, Spain; 2 Biobizkaia Health Research Institute, Pediatric Oncology, Barakaldo, Spain; 3 Spanish National Research Council (CSIC), Madrid, Spain; 4 LaboKCNQ, Barrio Sarriena, Leioa, Spain; 5 Biobizkaia Health Research Institute, Otolaryngology and Language and Communication Disorders, Barakaldo, Spain

**Keywords:** aging, ion channel, nervous system, neurodegeneration, neuroinflammation, sensory processing

Ion channels are complex macromolecular pores that regulate the ionic influx across cellular membrane. These proteins orchestrate the intracellular ionic homeostasis, necessary for life (D'Adamo et al., 2020). When the structural or functional integrity of these channels is compromised in the central nervous system (CNS), the resulting “channelopathies” provoke a broad spectrum of neurological disorders (Cerrone and Priori, 2012). This Research Topic explores the critical role of ion channels in maintaining the ionic balance or homeostasis and the pathological consequences of their malfunctioning.

## Ion channels in neuroinflammation and sensory processing

While historically ion channels function in focused on neuronal excitability, the field now recognizes the vital role of these channels in non-neuronal cells and in sensory integration. Sharma et al. provide a significant validation of the Hv1 voltage-gate proton channel function in BV2 microglial cells. Hv1 channels play an essential role in microglial functions, particularly under inflammatory conditions where their activity directly regulates the production of reactive oxygen species (ROS). Pharmacological inhibition of Hv1 channels during inflammation exerts neuroprotective effects. They used small molecule modulators to demonstrate that these channels are essential for managing the oxidative burst and pH homeostasis during neuroinflammatory response. This work emphasizes that ion channels are not just for excitable neurons; they are essential for the metabolic and immune functions of the brain resident macrophages. In this context, targeting Hv1 channels is emerging as a promising therapeutic strategy to alleviate neuroinflammation in neurodegenerative disorders and chronic pain, without causing severe consequences on normal physiological functions. Importantly, modulation of these channels does not completely abolish ROS production, thereby preserving cell viability. Moreover, their localization in the plasma membrane facilitates efficient drug-target interactions, supporting the development of effective pharmacological interventions.

Moving from the immune response to sensory processing, Chuinsiri et al., in a realm of sensory pathology, bridge the gap between research and clinics by examining the nociceptive and nociplastic spectrum of myofascial orofacial pain using studies utilizing models of direct stimulation or trauma to the masseter muscles. Their work illustrates how changes in ion channel expression drive the transition from acute nociceptive signaling to chronic. This research underscores that channelopathies are not always related to genetic defects but can result from maladaptive plasticity in the nervous system. However, further studies are required to clarify the degree to which these ion channels contribute to the maintenance of pain and to evaluate their potential as therapeutic targets. A major limitation of current research is that existing animal models and behavioral assays cannot reliably distinguish myogenous orofacial pain from other pain types, highlighting the lack of specific models for myofascial orofacial pain.

## Disruption in neurodegeneration and aging

Recent studies correlate neurodegeneration and ion channel dysfunction. The study of Maksour et al. highlights the multifaceted role of presenilin-1 in diverse cellular functions and underscores the need to clarify how specific mutations affect neuronal processes. Using iPSC-derived neurons modeling Alzheimer's Disease (AD), specifically focusing on two PSEN1 mutations, they find a paradoxical reduction in excitability, suggesting that early stages of AD are marked by profound ionic imbalance that precede several structural losses. This loss of function in neuronal firing patterns offers a new therapeutic landscape to broaden cognitive decline in neurodegenerative disorders. A clearer mechanistic understanding of the early alterations disrupted in AD will help identify the key processes that drive neurodegeneration and uncover new therapeutic strategies to slow the progression of this disorder. They propose that future studies could use genome-editing approaches to generate cell lines harboring PSEN1 mutations with varying degrees of pathogenicity to explore the potential link between neuronal excitability and the progression of early-onset AD.

Completing this, Urrutia et al. provide a comprehensive review of therapeutic role of voltage gate potassium (K_v_) channels in age-related neurodegenerative diseases. As primary regulators of resting membrane potential and action potential repolarization, K_v_ channels are necessary for preventing excitotoxicity. The authors suggest that pharmacological stabilization of K_v_ currents could serve as a neuroprotective strategy against the metabolic and oxidative stress associated with aging.

## Conclusion: a unified view of ion channel function and channelopathies

The contributions to this Research Topic reinforce the idea that ion channels are critical not only for neuronal excitability but also for microglial function and sensory processing, with dysfunction contributing to chronic pain and neurodegeneration From the regulation of microglial pH to the repolarization of aging neurons, these proteins are central for both health and disease. Thus, studies on Hv1, PSEN1, and Kv channels highlight that early ionic imbalances drive pathology, offering new therapeutic opportunities. The integration of specific modulators will be essential to move from describing channelopathies to treating them. Future research should develop specific models and assays, explore mutation-specific effects, and leverage pharmacological modulation to translate these mechanistic insights into targeted therapies that mitigate neuroinflammation, excitotoxicity, and cognitive decline.
